# Slower senescence in a wild insect population in years with a more female-biased sex ratio

**DOI:** 10.1098/rspb.2019.0286

**Published:** 2019-04-03

**Authors:** Rolando Rodríguez-Muñoz, Jelle J. Boonekamp, David Fisher, Paul Hopwood, Tom Tregenza

**Affiliations:** 1 Centre for Ecology and Conservation, School of Biosciences, University of Exeter, Penryn Campus, TR10 9FE, UK; 2 Groningen Institute for Evolutionary Life Sciences, University of Groningen, PO Box 11103, 9700 CC Groningen, The Netherlands; 3 Department of Psychology, Neuroscience and Behaviour, McMaster University, 6 Hamilton, Ontario, Canada

**Keywords:** ageing, senescence, cricket, *Gryllus*, life-history trade-off, sexual selection

## Abstract

Life-history theories of senescence are based on the existence of a trade-off in resource allocation between body maintenance and reproduction. This putative trade-off means that environmental and demographic factors affecting the costs of reproduction should be associated with changes in patterns of senescence. In many species, competition among males is a major component of male reproductive investment, and hence variation in the sex ratio is expected to affect rates of senescence. We test this prediction using nine years of demographic and behavioural data from a wild population of the annual field cricket *Gryllus campestris.* Over these generations, the sex ratio at adulthood varied substantially, from years with an equal number of each sex to years with twice as many females as males. Consistent with the predictions of theory, we found that in years with a greater proportion of females, both sexes experienced a slower increase in mortality rate with age. Additionally, phenotypic senescence in males was slower in years when there were more females. Sex ratio did not affect the baseline mortality rate in males, but females suffered higher age-independent mortality rates when males were in short supply.

## 1. Introduction

Alleles promote their continued existence in populations by conferring resilience on the organisms that carry them (survival), or by facilitating the production of new individuals carrying identical copies (reproduction). Alleles selected via either route can increase in overall representation, even if they are selected against via the alternative route, as long as the net effect is positive. Hence we should expect to see antagonistically pleiotropic alleles [1] that increase reproduction at the expense of decreasing lifespan (either directly or indirectly). This is the basis for adaptive life-history theories of senescence [2,3]. These theories posit that individuals trade-off investment in maintenance of their bodies against investment in reproduction, explaining why organisms decline in physiological performance and survival probability with age (senescence).

In most species, success in male–male competition for fertilizations is a key determinant of male reproductive success. Competition among males can involve direct interactions like sperm competition or fighting, or indirect interactions through sexual displays or calling to attract females for mating [4]. All such interactions involve energetic costs liable to influence the trade-off between survival and reproduction. Selection is expected to optimize the balance of investment between survival and reproduction in prevailing environmental and demographic conditions. This leads to the prediction that if individuals within a species are forced to increase reproductive investment by changes in prevailing conditions, they should show more rapid senescence. For example, where a higher proportion of males results in higher levels of male–male competition we would predict males should senesce faster than in generations where proportionately fewer males leads to less intense competition. This prediction does not appear to have been explicitly set out in the literature, but for males it is a straightforward consequence of the well-established relationship between sex ratio and the strength of sexual selection [5], and the putative trade-off between investment in reproduction and somatic maintenance [2,3,6]. For females predictions are less clear—in many species, females incur costs as a result of male competition for fertilizations [7,8], which would predict a similar covariation between population sex ratio and senescence. However, specific features of the ecology and mating system of individual species might impinge on how sex ratio affects costs of reproduction in females.

There is existing evidence showing that males which spend more energy in reproduction have reduced survival and senesce faster [9]. Laboratory studies of the effect of different levels of male–male coexistence have been carried out in invertebrates. Callander *et al*. [10] found that male field crickets housed with a rival had a reduced lifespan compared with those maintained on their own. In the fly *Telostylinus angusticollis*, variation in operational sex ratio resulted in later life increases in senescence for males in female-biased populations (apparently driven by inter-sexual interaction), but early life mortality increased and male lifespan was lower in male-biased populations [11]. Ideally, directly testing the influence of sex ratio on senescence would mean manipulating the sex ratio of a wild population and observing how rates of senescence vary. However, this would require either replicate populations, or for the manipulation to be repeated across a number of independent generations. In the absence of data from such a formidably challenging study, we aimed to exploit natural variation in sex ratio across generations of a population of wild crickets to test the prediction of an association between male bias in the sex ratio and more rapid actuarial and phenotypic senescence.

Over 10 years, we have closely monitored the survival and behaviour of a natural population of field crickets, *Gryllus campestris* in a meadow in northern Spain [12]. Each year we tag every individual in the population and by monitoring them for 24 h a day, using a network of video cameras, we have minute by minute measurements of phenotypic trait expression in both males and females and very precise demographic data [13].

**Figure 1.**
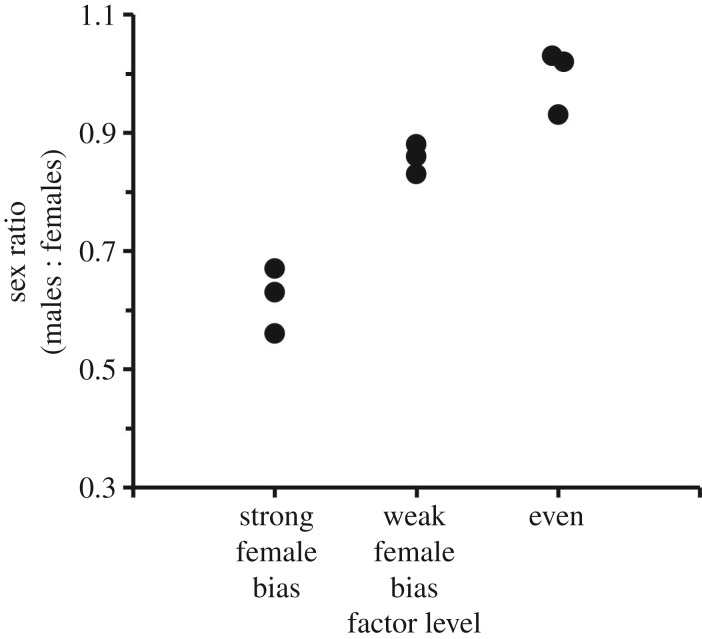
Values of the 3 years included in each sex ratio level of the factor used for the analysis of the relationship between sex ratio and senescence in wild *Gryllus campestris*.

In our population, adult sex ratio varies substantially among generations. Over the years of this study, the proportion of males relative to females ranged among generations from just over 0.5 (heavily female biased) to just around 1 (even) (figure 1; electronic supplementary material, table S1). Why this variation occurs is unknown. Crickets have chromosomal sex determination [14], so we assume a balanced sex ratio at laying. Juvenile males and females are superficially undifferentiated until genital development is visible in later instars and both sexes build and overwinter in apparently identical burrows.

Field cricket males experience significant reproductive competition [15], and there is significantly greater reproductive skew among males than females [12]. Male crickets engage in various energetically demanding reproductive behaviours including acoustic calling [16], fighting [17] and searching for females [18]. Therefore, we expect that males will need to invest more energy in years with a higher proportion of rival males. This leads to our prediction that male senescence will be more pronounced in these years.

## 2. Methods

### (a) Study system

We monitored a population of wild field crickets (*G. campestris)* in a meadow in northern Spain for 11 consecutive years (2006–2017). We are still processing the video from 2014 and 2017, and in 2006 we did not record temperature data so those years are not used in the present analysis. The meadow is managed in a similar way every year (for details of management and our monitoring regime, see [12,13]). By mid to late April, usually before the adults start to emerge, we install up to 133 infrared day/night cameras that record the activity around each burrow entrance continuously. Individuals of both sexes spend the vast majority of their time in this monitored area, or in their burrow, which is too narrow to allow matings or other interactions. A few days after emerging as an adult, we trap each individual. Each one is weighed (±0.01 g), photographed and marked with a PVC tag glued onto the pronotum, before being released back into the same burrow. The tag has a unique 1–2 character code (identity (ID)), which allows each individual to be identified on the video.

Since the number of occupied burrows is often greater than the number of cameras, and adult crickets regularly move around the meadow occupying different burrows, we carry out direct observations to cover non-videod burrows. We do this by directly observing the occupants of every burrow that lacks a camera every 1–2 days. We record the ID of any adult present or whether a nymph is in residence. This allows us to accurately record adult emergence dates even in burrows that are not directly monitored at that particular time, and means we are confident that it is very rare that we miss individuals. After the end of the season, we watch the videos and record all significant events (adult emergence, encounters between individuals, calling activity, matings, fights and their outcome, oviposition, predator attacks, movement of individuals around the meadow). A weather station installed in the centre of the meadow logs weather variables at 10 min intervals including measurements from seven additional temperature sensors at locations scattered around the meadow.

### (b) Measuring senescence

The two most common measures of senescence are based on the change in the probability of death with age (actuarial senescence), and on the decline in physical performance with age (phenotypic senescence). Actuarial senescence can be assessed from our direct observations of each individual adult once it has been tagged (capture–mark–recapture data). These include emergence date and either a direct observation of death or a date when the individual was last observed alive. To assess phenotypic senescence, we use our continuous observation data to provide measurements of changes in physically demanding traits with age.

### (c) Assessing differences in actuarial senescence in relation to sex ratio

We calculate sex ratio using the total number of males and females tagged each year. Since actuarial senescence is measured as a property of a cohort of individuals, the appropriate analytical approach is to define groups of interest and to compare patterns of senescence among these groups. We therefore divided our 9 years of data into three sex-ratio groups of 3 years each, representing even sex ratio years, weak female bias years and strong female bias years (figure 1). For each year, we designated the date when the first adult cricket emerged as the first day of that season. We then recorded the date of each cricket's emergence or first observation as an adult, and of each subsequent sighting of the cricket relative to that first date. We treated these observations as capture–recapture data and ran the analyses in BaSTA [19], an R package that estimates actuarial senescence, and re-sighting parameters using Bayesian analysis. There is a trade-off between achieving a high re-sighting probability at the expense of achieving a high resolution of birth and death events, i.e. the re-sighting probability will decrease when the survival observation bins are made smaller. Our video monitoring system provides continuous survival observations, which gives us the luxury of selecting an optimal balance between accuracy and resolution. We found that the re-sighting probability did not increase to a high value from 1-day to 5-days bins (51.3–70.4%, respectively) and, therefore, we prioritized the substantially higher resolution of 1-day bins achieved with a sufficient re-sighting probability. We used a Gompertz model with simple shape as it is the one that best fits our ageing data [13], including sex and the sex ratio group (see above) as grouping factors. The Gompertz model has two parameters; *b*_0_, the baseline mortality (the mortality rate independent of age), and *b*_1_, the age-dependent mortality rate (actuarial senescence); *b*_0_ is the intercept and *b*_1_ is the slope of a graph of age versus the natural log of the mortality rate. Positive values of *b*_1_ indicate that the probability of death increases with age. We ran four BaSTA simulations separately for each year, with 500 000 iterations, a burn-in parameter of 50 000 and a thinning rate of 2000, to achieve appropriate convergence and low serial auto-correlation (less than 0.1). To compare the posterior distributions between sexes, we used the Kullback–Leibler discrepancy calibration (KLDC) included in BaSTA [19]. This metric ranges from 0.5 to 1, with 0.5 indicating identical distributions, and 1 indicating completely different distributions. We considered KLDC values of ≥0.8 as indicative of differences between the two compared distributions [20–22].

### (d) Assessing male phenotypic senescence in relation to sex ratio

Previous analyses examined potential phenotypic senescence across a range of male traits [13], and identified male calling activity as the trait with the most robust evidence for senescence. In our study population, calling activity increases with age until it reaches a peak and declines subsequently [13]. We quantified calling activity for each male by recording whether he was calling or not over the first 10 min of every hour he was under observation. For those 10 min, at 1 min intervals we noted whether the male was calling or not. If any of those 10 samples was positive, then the cricket was recorded as calling in that hour. For each studied male, this measure provided up to 24 binary samples per day throughout its life (depending on how much his burrow was monitored). Only samples where the male was alone at the burrow and at least 5 days old were included (crickets take a few days before becoming sexually active after they emerge as adults). To reduce noise owing to small sample size, only days with five or more samples for any given male and males with at least 24 samples in total were included in the analysis.

In our study population, calling activity has a quadratic relationship with age. Males increase their calling activity over the first days after they become active, reach a peak and then start declining. We used the post-peak decline in calling to measure phenotypic senescence. We used the peak ages per year [13] to calculate the mean peak age of the 3 years within each of the two sex ratio groups, so that we could filter the pre-peak data and focus our analyses on the post-peak period. To allow us to directly compare phenotypic and actuarial senescence, we examined change in calling activity while including sex ratio as a categorical variable in the same way we did for the analysis of actuarial senescence. We decomposed age into delta (*Δ*) and mean (*μ*) ages according to Van de Pol & Wright [23]. This allows us to separate within-individual effects from among-individual effects, i.e. it separates the effect of age on calling effort within individuals from any potential age-related selective mortality (differences among males due to differences in the age when their calling is measured). We ran a mixed model using the *lme4* R package [24], and included calling (*Sings*) as a binary response trait scored as 1 if the cricket was calling at any given sampled time or 0 if he was not. We included ambient temperature (*Temp*), mean age (*μAge*) and the interaction between within-individuals age and sex ratio (ΔAge*SR) as fixed effects, and cricket identity (*ID*) and year (*Year*) as random effects.

## 3. Results

### (a) Differences in actuarial senescence in relation to sex and sex ratio

**Figure 2.**
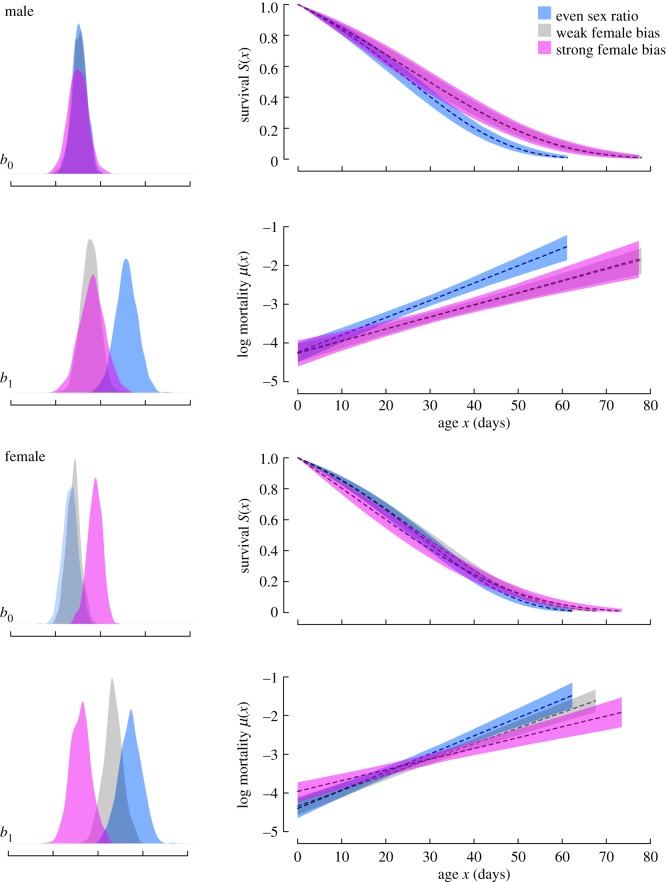
Posterior distributions for the 95% credible intervals of baseline mortality (*b*_0_) and age-dependent mortality (*b*_1_) in wild *Gryllus campestris* comparing between years with contrasting sex ratios (figure 1). All parameters were estimated using the BaSTA R package [19] using a Gompertz model with simple shape. Within sexes, females have lower *b*_0_ in even sex ratio years, and both have a lower *b*_1_ in years with strong female bias. Between sexes, *b*_1_ is similar for males and females under even and strong female bias, and shows an intermediate value in females under weak female bias. Females have higher *b*_0_ in years with strong female bias. (Online version in colour.)

Both sexes showed higher age-dependent mortality (*b*_1_) under even sex ratios compared to female-biased sex ratios, i.e. they senesced faster when the proportion of males in the population was higher. When comparing between the sexes, age-dependent mortality showed a nearly perfect overlap for males and females within the two extremes of the sex ratio levels, although females showed a higher value under weak female-biased sex ratio. Baseline mortality (*b*_0_) did not differ between sex ratio levels in males, but in females it was higher when the proportion of females was highest (figure 2 and table 1).

**Table 1.** Estimates and 95% credible intervals of baseline mortality rate (*b*_0_, the mortality independent of age) and age-dependent mortality rate (*b*_1_, the coefficient for the effect of age on mortality) for each sex in a wild population of *Gryllus campestris*. (Each row shows the pattern of variation when data are divided into three groups of three generations, each group representing three levels of sex ratio (figure 1). Scores for the Kullback–Leibler divergence criterion (KLDC) between sexes and sex ratio levels are included (values > 0.8 are highlighted in italics). Estimates were calculated using BaSTA [19] and a Gompertz model with simple shape. For each parameter, the within sex KLDC values correspond to the comparison between contiguous sex ratio levels, with the only exception of the lower one, which compares the two extreme levels.)

parameter	level	males	females	KLDC
*b*_0_ baseline mortality	strong female bias	−4.263 (−4.602, −3.932)	−3.962 (−4.235, −3.727)	*0*.*936*
	KLDC	0.546	*0.994*	
	weak female bias	−4.250 (−4.497, −4.017)	−4.338 (−4.568, −4.098)	0.613
	KLDC	0.503	0.555	
	even	−4.235 (−4.495, −3.997)	−4.398 (−4.648, −4.144)	0.768
	KLDC	0.546	*0.998*	
*b*_1_ age-dependent mortality	strong female bias	0.031 (0.021, 0.040)	0.027 (0.020, 0.035)	0.648
	KLDC	0.557	*0.998*	
	weak female bias	0.030 (0.023, 0.037)	0.040 (0.032, 0.047)	*0*.*988*
	KLDC	*0.999*	*0.880*	
	even	0.044 (0.035, 0.052)	0.046 (0.038, 0.055)	0.578
	KLDC	*0.992*	*1.000*	

### (b) Effect of sex ratio on male phenotypic senescence

**Figure 3.**
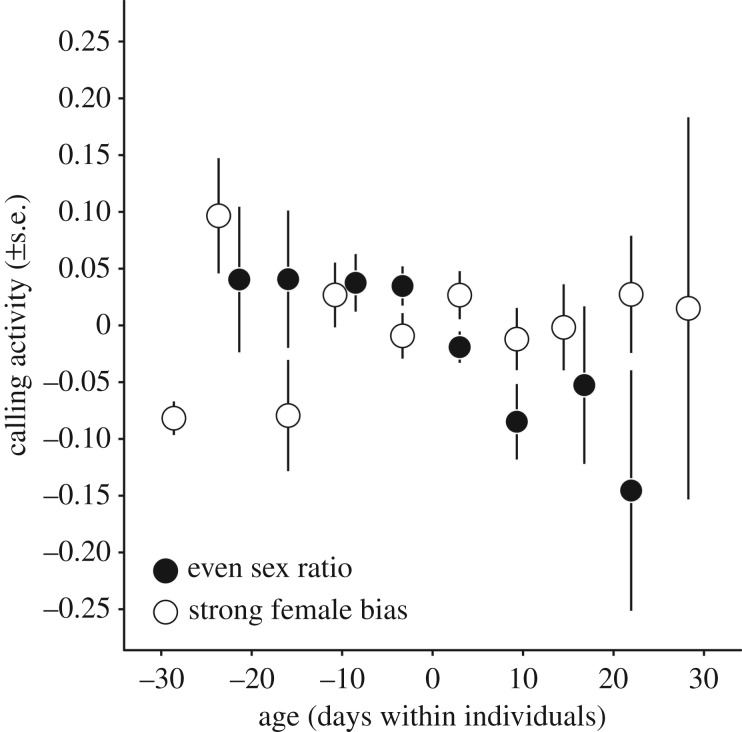
Within individual age trajectories of male calling activity comparing the two most extreme sex ratio groups: the 3 years with an even sex ratio (black circles) and the 3 years with most female-biased sex ratio (open circles). Data points and error bars reflect the mean calling activity of age bins and their respective standard errors (note that the statistical analyses were done with the raw data, i.e. without binning of age). In years with a higher ratio of males : females calling activity declines as males age, whereas this decline is absent in strong female-biased years. We omitted the intermediate sex ratio years for reasons of clarity—data for all 9 years are available in table 2.

There was a highly significant interaction between sex ratio level and the decline in calling activity with age; males experienced a fast decline in calling when the number of males and females was similar, but showed no decline in years where females outnumbered males (figure 3 and table 2).

**Table 2.** Analysis of the interaction between sex ratio (*SR*), and the relationship between age and the probability of calling (*Sings*) in wild *Gryllus campestris* males after the age when the cricket population reaches its maximum in calling activity*.* (Sex ratio was classified in three levels ranging from strongly female biased to even (figure 1). Each level included three generations (years). We decomposed age into delta age (*ΔAge*), representing within individuals effects and mean age (*μAge)*, representing among individuals effects (*Age* = *μAge* + *ΔAge* [23]. We included ambient temperature (*Temp*) when each calling sample was recorded, the interaction between *ΔAge* and *SR* and mean age (*μAge*) as fixed effects, and individual identity (*ID*) and year (*Year*) as random effects. The table shows the results of a mixed model using the *lme4* R package with a binomial error distribution (*Sings ∼ Temp + ΔAge + ΔAge:SR + μAge + (1|ID) + (1|Year)*). Coefficients with significant *p*-values are highlighted in bold.)

fixed effects	coeff.	s.d.	*p*
*Intercept*	−5.413	0.164	**<0.001**
*Temp*	0.284	0.004	**<0.001**
*ΔAge*	−0.245	0.036	**<0.001**
*weak female-biased SR*	−0.153	0.214	0.476
*strong female-biased SR*	−0.089	0.222	0.687
*μAge*	0.058	0.065	0.365
*ΔAge: weak female-biased SR*	0.140	0.041	**0.001**
*ΔAge: strong female-biased SR*	0.250	0.046	**<0.001**
**samples**	53 171		
**random effects**	**variance**	**s.d.**	N
*ID*	0.434	0.659	327
*Year*	0.053	0.230	9

## 4. Discussion

Our findings are compatible with the prediction that males senesce at a faster rate in years when the level of competition among males is greater. We find that in years with proportionately more males, males have both an accelerated increase in mortality with age and a more rapid decline in their calling activity. Life-history trade-off theories of ageing predict exactly this association between environmental and demographic factors that influence the extent to which males are involved in reproductive competition and subsequent senescent declines [6,25–27].

Because we did not directly manipulate sex ratio, we cannot unequivocally establish sex ratio as the direct cause of the observed differences in senescence rate. An alternative explanation is that senescence is directly impacted by the same environmental factors that affect male survival resulting in sex ratio variation. These might range from climatic conditions to variation in the prevalence of unidentified sex ratio distorting parasites, and could have diverse modes of action. For instance, there might be a carryover effect if harsh winter conditions disproportionately affect male survival: in years with fewer males, those that do survive to adulthood may be in generally poorer condition [28]. Conversely, harsh overwintering conditions could mean that only the highest quality males reach adulthood, increasing average male condition. Even if we were able to distinguish among possible alternative scenarios, our predictions would not be clear, for instance, an experimental study in the fly *T. angusticollis* Hopper *et al.* [29] found that poor condition males senesced at a slower rate than high condition males.

Another potential explanation for the pattern we observe would be the existence of an effect of population density rather than sex ratio. In this scenario, the intensity of intra-sexual competition would be owing mainly to the absolute number of males rather than the proportion of males relative to females. To assess this possibility, we analysed the combined effect of sex ratio and population density as continuous variables on how calling activity changes with age, including all the years in the analysis. We found that the three-way interaction was not significant, i.e. there was no combined effect of sex ratio and population density on the relationship between age and calling activity post-peak. Increasing density showed a positive effect on the relationship between calling activity and age, but the negative effect of sex ratio on that relationship remained significant independent of population density (see the electronic supplementary material, table S2).

Our previous work provides some evidence for a relationship between increased early life reproductive effort and subsequent more rapid phenotypic senescence in males [30]. If intra-sexual competition is the reason for the increase in senescence rate that we observe in years with relatively few females, we might expect that both sex ratio and population size would predict male reproductive effort (see the electronic supplementary material). Opposite to this prediction, we found that both sex ratio and population density had a negative effect on calling effort, i.e. males called less when the proportion of males or population density increased, although the interaction between sex-ratio and population density was negative (electronic supplementary material, table S3). Neither the intensity of searching activity nor dominance in fights were related to sex ratio or population density (electronic supplementary material, tables S4 and S5). These findings are difficult to interpret; male reproductive effort will be composed of numerous aspects of the phenotype, many of which will be cryptic. For instance, it is easy to imagine that when males are less common it is a better strategy to sing more, because there are more females per male that might be attracted, but more cryptic aspects of male reproductive investment may be reduced.

Our finding that females also showed slower senescence in female-biased years might reflect costs of higher rates of interaction with males in years when males are more common, as hypothesized to explain a similar pattern in the fly *T. angusticollis* [11]. Our observation of higher baseline female mortality in years when females outnumbered males is consistent with our earlier observation of females gaining protection from predation by sharing a burrow with a male [31]: because it is extremely rare for a male to share his burrow with more than one female, when females are in excess there will be a proportion of the female population that is unable to seek protection by sharing a burrow. Alternatively, females may need to search harder for males when they are relatively scarce which might expose them to greater predation risks. Either of these effects would explain the difference in baseline mortality affecting only females that we observe.

In conclusion, we show that senescence rate in males is closely related to sex ratio, increasing in more male-biased years as predicted by life-history trade-off theories of senescence. We lack direct evidence that this relationship is driven by intra-sexual competition because of the caveat that there is always the potential for relationships between natural variation in sex ratio and other factors that might also affect senescence. Future studies in which sex ratio is manipulated across multiple generations or across multiple populations are the next step to provide further insights into potential trade-offs between reproduction and senescence. Our study demonstrates that a significant proportion of the variation in senescence rate in the wild can be explained by variation in environmental factors. The strength of these effects suggests that the effort needed to execute an experimental investigation into the mechanistic basis of our observations would be worthwhile.

## Supplementary Material

Additional analyses including four tables

## Data accessibility

Data available from the Dryad Digital Repository: https://doi.org/10.5061/dryad.fk52454 [32]. Files include (1) capture-recapture dataset and (2) data on phenotypic senescence.
