# Supplementary material for: Slower senescence in a wild insect population in years with a more female-biased sex ratio
Source: Proc Biol Sci. 2019 Apr 3;286(1900):20190286. doi: 10.1098/rspb.2019.0286 (PMC6501688; doi:10.1098/rspb.2019.0286)
Supplement: Additional analyses including four tables [file rspb20190286supp1.docx]

### Supplementary material from “Slower senescence in a wild insect population in years with a more female-biased sex ratio”

Rolando Rodríguez-Muñoz, Jelle J. Boonekamp, David Fisher, Paul Hopwood and Tom Tregenza

DOI: 10.1098/rspb.xxxx.xxxx

## Effect of sex ratio and population density on the relationship between calling activity and age

We analysed the combined effect of sex ratio and population density (varying across years, see Table S1) on the relationship between calling activity and age during the period after males reach their peak of calling activity (post-peak). We used the mean of the peak ages across the 9 years included in the study (15 days). We ran a mixed model using the *lme4* R package (Bates et al. 2014), with calling as a binary response variable (whether a male was calling (1) or not (0) every time he was sampled). We included temperature and the interaction between age within individuals, yearly sex ratio and population density as fixed effects, and individual identity as a random effect. The interaction was negative between age and sex ratio, but positive between age and population density. There was no three-way interaction between age, sex ratio and population density (see Table S2).

Table S1. Population size and sex ratio per year.

| **Year** | **2007** | **2008** | **2009** | **2010** | **2011** | **2012** | **2013** | **2015** | **2016** |
| --- | --- | --- | --- | --- | --- | --- | --- | --- | --- |
| **Population** | 198 | 67 | 154 | 185 | 199 | 100 | 239 | 103 | 108 |
| **Sex ratio** | 0.83 | 0.63 | 0.86 | 0.67 | 0.88 | 0.56 | 1.03 | 1.02 | 0.93 |

Table S2. Analysis of the probability of calling in relation to age, sex ratio and population density from nine years of data in wild *Gryllus campestris* males. We included only the portion of the crickets’ lives after the peak in calling activity, when it tends to decline. We decomposed age into delta age (*ΔAge*), representing within individuals effects, and mean age (*µAge*), representing among individuals effects (*Age* = *µAge + ΔAge*, (see Van de Pol and Wright 2009)). We included the ambient temperature (*Temperature*) when each calling sample was recorded, the interaction between delta age, sex ratio and population density (*ΔAge*SexRatio*PopDens*) and mean age (*µAge*) as fixed effects, and individual identity (*ID*) as a random effect. The table shows the results of a mixed model using the *lme4* R package (Bates et al. 2014) with a binomial error distribution. Coefficients with significant P values are highlighted in bold italics.

| **Fixed effects** |  | **Coeff.** | **SD** | **P** |
| --- | --- | --- | --- | --- |
|  |  |  |  |  |
| *Intercept* |  | -5.466 | 0.073 | ***< 0.001*** |
| *Temperature* |  | 0.283 | 0.004 | ***< 0.001*** |
| *ΔAge* |  | -0.126 | 0.016 | ***< 0.001*** |
| *SexRatio* |  | -0.020 | 0.043 | 0.646 |
| *PopDens* |  | -0.160 | 0.043 | ***< 0.001*** |
| *µAge* |  | 0.053 | 0.060 | 0.378 |
| *ΔAge*SexRatio* |  | -0.089 | 0.020 | ***< 0.001*** |
| *ΔAge* PopDens* |  | 0.075 | 0.020 | ***< 0.001*** |
| *SexRatio* PopDens* |  | -0.097 | 0.034 | ***0.004*** |
| *ΔAge*SexRatio* PopDens* |  | 0.026 | 0.015 | 0.091 |
|  |  |  |  |  |
| **Samples** |  | 53,171 |  |  |
| **Random effects** |  | **Variance** | **SD** | **N** |
| *ID* |  | 0.445 | 0.667 | 327 |

## Relationship between reproductive effort and sex ratio and population density

Our prediction was that males under stronger intra-sexual competition would senesce faster because of an increase in their expenditure in reproductive traits at a cost to body maintenance. If this prediction is correct and both sex ratio and population density increase male-male competition, we would expect that both variables will be positively associated with reproductive effort. To test this prediction, we measured individual reproductive effort early in adult life (before the peak in calling activity, see above), and analysed how it relates to among-years variation in sex ratio and population size. We measured reproductive effort based on three traits, calling effort, searching activity and dominance in fights. These traits are known to be energetically costly (Hoback and Wagner 1997) or have shown some evidence of having a positive relationship to senescence rate in late life (Rodriguez-Muñoz et al. In Press).

## Measures of reproductive effort (modified from Rodriguez-Muñoz et al. In Press)

***Calling activity***: Male crickets sing from outside their burrows to attract potential mates. We quantified calling activity for each male by recording whether he was singing or not over the first 10 minutes of every hour. For those ten minutes, at one minute intervals we noted whether the male was singing or not. If any of those ten samples was positive, then the cricket was recorded as singing in that hour. If singing was not observed for any of the ten samples, he was recorded as not singing. For each studied male, this measure provided up to 24 binary samples per day throughout its life (depending on how much his burrow was monitored). We only included samples where the male was alone at the burrow and at least five days old. To reduce noise due to small sample size, only days with five or more samples for any given male and males with at least 24 samples in total were included in the analysis. For each male, we quantified individual reproductive effort pre-peak of calling activity as the percentage of samples when a male was found calling.

***Searching activity***: Male crickets also find potential mates by visiting different burrows around the meadow. To score the intensity of this searching activity, we used the duration of visits that each individual made to burrows; shorter stays are associated with more frequent moves between burrows. The duration of burrow visits is extremely right skewed, ranging from less than 1 min to over 10 days with a median of 77 min. To analyse these skewed data, we transformed searching activity into a binary variable by classifying burrow stays as either short (≤ 77 min, representing intensive searching) or long (> 77 min, representing lower searching activity). We coded short stays as 1 and long stays as 0 in order to place more actively searching males in the high scoring group for consistency with the other measures. Because stay duration can be influenced by the presence of another cricket at the same burrow, we only included visits where the focal male was on his own for the duration of the whole visit. Using the available date pre-peak, we estimated how active was each individual male as his proportion of short stays, and used this as the response variable. The proportion of short stays has strongly bimodal distribution. To facilitate the statistical analysis, we calculated the median proportion across all males and scored each of them as a low activity male (0) if his proportion was below the median or a high activity male (1) if he was above the median.

***Dominance in fights***: Males frequently encounter other males at burrows. When this happens, either one of them leaves the area immediately, or they engage in a fight (typically lasting for only a few seconds) for the occupation of the burrow. Burrow occupancy allows the male the opportunity to mate with any female using the burrow, so a male’s success in these fights is a key measure of his dominance. Although each fight only produces a single independent piece of data (there is always a winner and a loser) there are many more fights than there are males. Similarly to what we did for searching activity, we estimated how dominant a male was as the proportion of fights won. However, because this variable also has a strong bimodal distribution, we followed the same procedure used with the previous variable of calculating the median dominance across all males and scoring each of them as dominant (1) or subordinate (0).

## Statistical analyses

We analysed the relationship between reproductive effort and sex ratio and population density by running general linear models in R, including either calling effort, searching activity or dominance as the response variable, and the interaction between sex ratio and population density as fixed effects. For calling effort, we took the square-root and rounded the values with no decimals to use a Poisson family distribution. For searching and dominance, we used a binomial family distribution.

## Effect of sex ratio and population density on reproductive effort

Both, sex ratio and population density had a negative effect on calling effort, i.e., males called less when the proportion of males or population density increased, although the interaction between sex ratio and population density was negative (Table S3). Neither the intensity of searching activity nor dominance in fights were related to sex ratio or population density (Tables S4 and S5).

Table S3. Analysis of the relation between the intensity of calling activity in early adult life, and the interaction between the yearly values of sex ratio (*SexRatio*) and population size (*PopDens*) over nine years*.* We used general linear models (glm) in R, including the intensity in calling activity pre-peak for each individual male as the response variable, the annual values of sex ratio and population density as fixed effects, and a Poisson family. Coefficients with significant *P* values are highlighted in bold italics.

| **Fixed effects** |  | **Coeff.** | **SD** | **P** |
| --- | --- | --- | --- | --- |
|  |  |  |  |  |
| *Intercept* |  | 0.715 | 0.046 | ***< 0.001*** |
| *SexRatio* |  | -0.168 | 0.051 | ***0.001*** |
| *PopDens* |  | -0.210 | 0.049 | ***< 0.001*** |
| *SexRatio*PopDens* |  | -0.144 | 0.042 | ***< 0.001*** |
|  |  |  |  |  |
| **Samples** |  | 253 |  |  |

Table S4. Analysis of the relationship between the intensity of searching activity in early adult life, and the interaction between the yearly values of sex ratio (*SexRatio*) and population size (*PopDens*) over nine years*.* We used general linear models (glm) in R, including the intensity of searching activity pre-peak for each individual male as the response variable, the annual values of sex ratio and population density as fixed effects, and a binomial family distribution. Coefficients with significant *P* values are highlighted in bold italics.

| **Fixed effects** |  | **Coeff.** | **SD** | **P** |
| --- | --- | --- | --- | --- |
|  |  |  |  |  |
| *Intercept* |  | -0.001 | 0.120 | 0.999 |
| *SexRatio* |  | -0.082 | 0.132 | 0.536 |
| *PopDens* |  | 0.162 | 0.128 | 0.207 |
| *SexRatio*PopDens* |  | -0.090 | 0.092 | 0.328 |
|  |  |  |  |  |
| **Samples** |  | 314 |  |  |

Table S5. Analysis of the relationship between male dominance early adult life, and the interaction between the yearly values of sex ratio (*SexRatio*) and population size (*PopDens*) over nine years*.* We used general linear models (glm) in R, including dominance pre-peak for each individual male as the response variable, the annual values of sex ratio and population density as fixed effects, and a binomial family. Coefficients with significant *P* values are highlighted in bold italics.

| **Fixed effects** |  | **Coeff.** | **SD** | **P** |
| --- | --- | --- | --- | --- |
|  |  |  |  |  |
| *Intercept* |  | -0.080 | 0.157 | 0.609 |
| *SexRatio* |  | -0.044 | 0.177 | 0.801 |
| *PopDens* |  | -0.197 | 0.164 | 0.231 |
| *SexRatio*PopDens* |  | -0.131 | 0.110 | 0.232 |
|  |  |  |  |  |
| **Samples** |  | 172 |  |  |

## References

Bates, D., M. Mächler, B. Bolker, and S. Walker. 2014. Fitting linear mixed-effects models using lme4. arXiv preprint arXiv:1406.5823.

Hoback, W. W. and W. E. Wagner. 1997. The energetic cost of calling in the variable field cricket, *Gryllus lineaticeps*. Physiol. Entomol. 22:286-290.

Rodriguez-Muñoz, R., J. J. Boonekamp, L. Xingping, I. Skicko, D. N. Fisher, P. E. Hopwood, and T. Tregenza. In Press. Testing the effect of early life reproductive effort on age-related decline in a wild insect. Evolution.

Van de Pol, M. and J. Wright. 2009. A simple method for distinguishing within-versus between-subject effects using mixed models. Anim. Behav. 77:753-758.
